# Supplementary material for: Low Physical Performance Could Be Associated with Adverse Health Outcomes over Time: Results from a Cohort of Older Adults
Source: Int J Environ Res Public Health. 2024 Mar 9;21(3):319. doi: 10.3390/ijerph21030319 (PMC10969904; doi:10.3390/ijerph21030319)
Supplement: Supplementary file 1 [file ijerph-21-00319-s001.zip › ijerph-2790351-supplementary.pdf]

Table S1: Physical performance tests according to demographic, socioeconomic, behavioral and health-related characteristics.

	Low Gait Speed (baseline)[a]		Declined GS (2014-2019)[b]		Low PP by TUG (baseline)[c]		Worsening TUG (2014-2019)[b]	
**Variables**	N* 1305	*p-value*[d] % (95%CI)	N** 476	*p-value*[d] % (95%CI)	N* 1433	*p-value*[d] % (95%CI)	N** 471	*p-value*[d] % (95%CI)
**Sex**		***0.001***		*0.099*		***0.003***		*0.256*
Female	68	22.3 (18.0; 27.4)	198	65.1 (59.6; 70.3)	99	32.4 (27.3; 37.8)	118	71.5 (64.1; 77.9)
Male	16	9.7 (6.0; 15.3)	120	72.3 (65.4; 79.0)	32	19.4 (14.0; 26.2)	203	66.3 (60.8; 71.4)
**Age (completed years)**		***<0.001***		*0.926*		***<0.001***		*0.329*
60-69	34	11.9 (8.06 16.2)	195	68.2 (62.5; 73.3)	55	19.3 (15.1; 24.3)	189	66.3 (60.6; 71.6)
70-79	29	19.7 (14.1; 27.0)	98	66.7 (58.6; 73.8)	50	33.6 (26.4; 41.5)	103	69.1 (61.2; 76.0)
80+	21	58.3 (41.9; 73.1)	25	69.4 (52.7; 82.2)	26	70.3 (53.8; 82.7)	29	78.4 (62.3; 88.8)
**Skin color**		*0.277*		*0.525*		*0.895*		*0.899*
White	65	17.0 (13.5; 21.1)	257	67.1 (62.2; 71.6)	106	27.6 (23.4; 32.3)	261	68.0 (63.1; 72.5)
Other than white	19	22.1 (14.6; 32.1)	61	70.9 (60.5; 79.6)	25	28.7 (20.2; 39.1)	60	69.0 (58.5; 77.8)
**Marital status**		***0.008***		*0.152*		***0.002***		*0.294*
Married/with a partner	42	14.8 (11.1; 19.5)	197	69.6 (64.0; 74.7)	67	23.8 (19.1; 29.1)	196	69.5 (63.9; 74.6)
Without a partner/separated	10	13.9 (7.6; 24.0)	52	72.2 (60.8; 81.3)	16	22.2 (14.1; 33.3)	52	72.2 (60.8; 81.3)
Widow(er)	32	28.1 (20.6; 37.0)	69	60.5 (51.3; 69.1)	48	41.0 (32.5; 50.2)	73	62.4 (53.3; 70.7)
**Economic level[e]**		***0.001***		*0.216*		*0.136*		*0.934*
A/B	18	11.2 (7.1; 17.1)	115	71.4 (64.0; 77.9)	38	23.3 (17.4; 30.4)	109	66.9 (59.3; 73.7)
C	47	19.4 (14.9; 24.9)	159	65.7 (59.5; 71.4)	72	29.8 (24.3; 35.8)	166	68.6 (62.5; 74.1)
D/E	16	34.8 (22.5; 49.5)	27	58.7 (44.1; 71.9)	17	37.0 (24.3; 51.7)	31	67.4 (52.7; 79.3)
**Education level (completed years)**		***<0.001***		*0.288*		***<0.001***		*1.000*
None	15	27.8 (17.5; 41.1)	36	66.7 (53.1; 77.9)	25	45.5 (32.9; 58.7)	38	69.1 (55.7; 79.9)
1-7	58	22.0 (17.4; 27.4)	172	65.2 (59.2; 70.7)	79	29.7 (24.5; 35.5)	181	68.1 (62.2; 73.4)
≥8	10	6.7 (3.6; 12.0)	109	72.7 (65.0; 79.2)	27	18.1 (12.7; 25.2)	101	67.8 (59.9; 74.8)
**Current work situation**		***0.002***		*1.000*		***0.023***		*0.467*
Unemployed	71	21.0 (17.0; 25.7)	229	67.8 (62.6; 72.5)	104	30.5 (25.8; 35.6)	66	65.4 (64.1; 73.9)
Employed	8	7.8 (4.0; 14.9)	69	67.7 (58.0; 76.0)	19	18.8 (12.3; 27.7)	236	69.2 (55.6; 74.0)

**Diet quality[f]**		*0.480*		*0.878*		*0.539*		*0.79*
Low	29	20.7 (14.8; 28.2)	97	69.3 (61.1; 76.4)	43	30.7 (23.6; 38.9)	92	65.7 (57.5; 73.1)
Average	32	18.3 (13.2; 24.7)	117	66.9 (59.5; 73.4)	49	28.0 (21.8; 35.1)	121	69.1 (61.9; 75.6)
High	23	15.2 (10.3; 21.9)	101	66.9 (59.0; 73.9)	38	24.8 (18.6; 32.3)	105	68.6 (60.8; 75.5)
**Leisure-time physical activity[g]**		*0.128*		*1.000*		*0.245*		*0.900*
≤150 min/week	73	19.6 (15.9; 24.0)	253	68.0 (63.1; 72.6)	109	29.2 (24.8; 34.1)	255	68.4 (63.5; 72.9)
>150 min/week	11	12.1 (6.8; 20.6)	62	68.1 (57.9; 76.9)	21	22.8 (15.4; 32.5)	64	69.6 (59.4; 78.1)
**Smoking**		*0.065*		*0.626*		*0.053*		*0.624*
Not a smoker	55	21.4 (16.8; 26.9)	179	69.7 (63.7; 75.0)	83	31.9 (26.5; 37.9)	177	68.1 (62.1; 73.5)
Smoker	10	17.6 (9.7; 29.7)	38	66.7 (53.5; 77.6)	10	17.5 (9.7; 29.7)	36	63.2 (50.0; 74.6)
Former smoker	19	12.3 (7.9; 18.4)	101	65.2 (57.3; 72.3)	38	24.7 (18.5; 32.1)	108	70.1 (62.4; 76.8)
**Alcohol consumption[h]**		*0.052*		*0.257*		*0.059*		*0.790*
No	70	20.0 (16.1; 24.5)	232	66.3 (61.2; 71.1)	106	30.1 (25.5; 35.1)	238	67.6 (62.5; 72.3)
Yes	14	11.8 (7.1; 18.9)	86	72.3 (63.5; 79.6)	25	21.0 (14.6; 29.3)	83	69.8 (60.9; 77.3)
**Multimorbidity**		***0.001***		*0.101*		***<0.001***		*0.471*
Up to 4 diseases	18	10.3 (6.60; 15.8)	110	63.2 (55.8; 70.1)	31	17.8 (12.8; 24.2)	115	66.1 (58.7; 72.8)
5 or more diseases	65	22.7 (18.2; 28.0)	202	70.6 (65.1; 75.6)	99	34.3 (29.0; 39.9)	201	69.6 (64.0; 74.6)
**Depression[i]**		***0.001***		*1.000*		***0.002***		*0.565*
No	61	15.2 (12.0; 19.1)	271	67.6 (62.8; 72.0)	100	24.9 (20.9; 29.3)	273	67.9 (63.2; 72.3)
Yes	22	34.4 (23.8; 46.8)	43	67.2 (54.8; 77.6)	29	44.6 (33.0; 56.8)	47	72.3 (60.2; 81.8)
**Polypharmacy[j]**		***<0.001***		*1.000*		***<0.001***		*0.338*
0-4 medications	45	30.6 (23.7; 38.6)	100	68.0 (60.0; 75.1)	59	39.9 (32.3; 48.0)	96	64.9 (56.8; 72.1)
≥5 medications	39	12.1 (9.0; 16.2)	218	67.7 (62.4; 72.6)	72	22.3 (18.1; 27.2)	225	69.7 (64.4; 74.4)
**BMI[k]**		*0.897*		*0.479*		*0.924*		*0.492*
<22.0 kg/m²	5	19.2 (8.2; 38.8)	16	61.5 (42.0; 77.9)	8	30.8 (16.2; 50.6)	15	57.7 (38.5; 74.8)
22.0-27.0 kg/m²	28	18.8 (13.3; 25.9)	106	71.1 (63.3; 77.9)	41	27.0 (20.5; 34.6)	105	69.1 (61.3; 75.9)
>27.0 kg/m²	51	17.5 (13.6; 22.3)	194	66.7 (61.0; 71.9)	81	27.8 (23.0; 33.3)	201	69.1 (63.5; 74.1)

*corresponding to the total sample that performed the physical tests at baseline (2014)

**corresponding to the number of individuals who performed the physical tests in 2014 and 2019/20

[a] acoording to cutoff points recommended by the European Working Group on Sarcopenia in Older People 2 (2019).

[b] declined GS and increased TUG timed = declined physical performance. Clinically relevant changes were defined as a variation ≥0.1 m/s in the GS and ≥5% in the TUG time from 2014 to 2019-20.

[c] cutoff points according to sample distribution.

[d] Fisher´s exact test.

[e] according to *Associação Brasileira de Empresas de Pesquisa* (ABEP): category A/B indicating higher socioeconomic status;

[f] assessed using the Diet Quality Index for the Elderly (*Índice de Qualidade da dieta do idoso* - IDQ-I);

[g] assessed by the International Physical Activity Questionnaire (IPAQ);

[h] alcohol consumption in the last month;

[h] according to the Geriatric Depressive Scale (GDS-10);

[j] continuous use of medications;

[k] cutoff points recommended by Lipschitz et al.

Table S2: Baseline Gait Speed (GS) and Timed Up and Go (TUG) and change in both tests from 2014 to 2019 in older adults according according to the place of consultation with a health professional in the last year.

Variables	Place of consultation (specialized care + emergency service)[a] N=348	
	Crude *p-value* PR (95%CI)	Adjusted *p-value*[b] PR (95%CI)
**GS (m/s) in 2014[c] (n=1305)**	*0.574*	*0.599*
Normal (>0.8 m/s) (n=994)	1.00	1.00
Low (≤0.8 m/s) (n=311)	0.98 (0.92; 1.05)	1.02 (0.95; 1.09)
**Change in GS (2014-2019)[d] (n=476)**	*0.992*	*0.790*
Stability-Improvement (n=152)	1.00	1.00
Decline (n=324)	1.00 (0.95; 1.05)	1.01 (0.96; 1.06)
**TUG (s) in 2014[e] (n=1433)**	*0.367*	*0.067*
Normal (≤11 s) (n=1231)	1.00	1.00
Low performance (>11 s) (n=202)	1.02 (0.97; 1.08)	1.05 (1.00; 1.11)
**Change in TUG (2014-2019)[d] (n=471)**	*0.193*	*0.331*
Stability-Improvement (n=150)	1.00	1.00
Worsening (n=321)	0.97 (0.92; 1.02)	0.98 (0.93; 1.02)

[a] this variable was dichotomized into: primary care consultation (reference group; N=126) and specialized care + emergency service (N=348)

[b] p-value obtained through robust Poisson regression including variables according to the DAG model: economic level, diet quality, physical activity, multimorbidity, depression, polypharmacy, and BMI.

[c] cutoff point recommended by the European Working Group on Sarcopenia in Older People 2 (2019).

[d] clinically relevant changes were defined as a variation of ≥0.1 m/s in GS and ≥5% in TUG time.

[e] cutoff points according to sample distribution: lower tertile defines low performance.

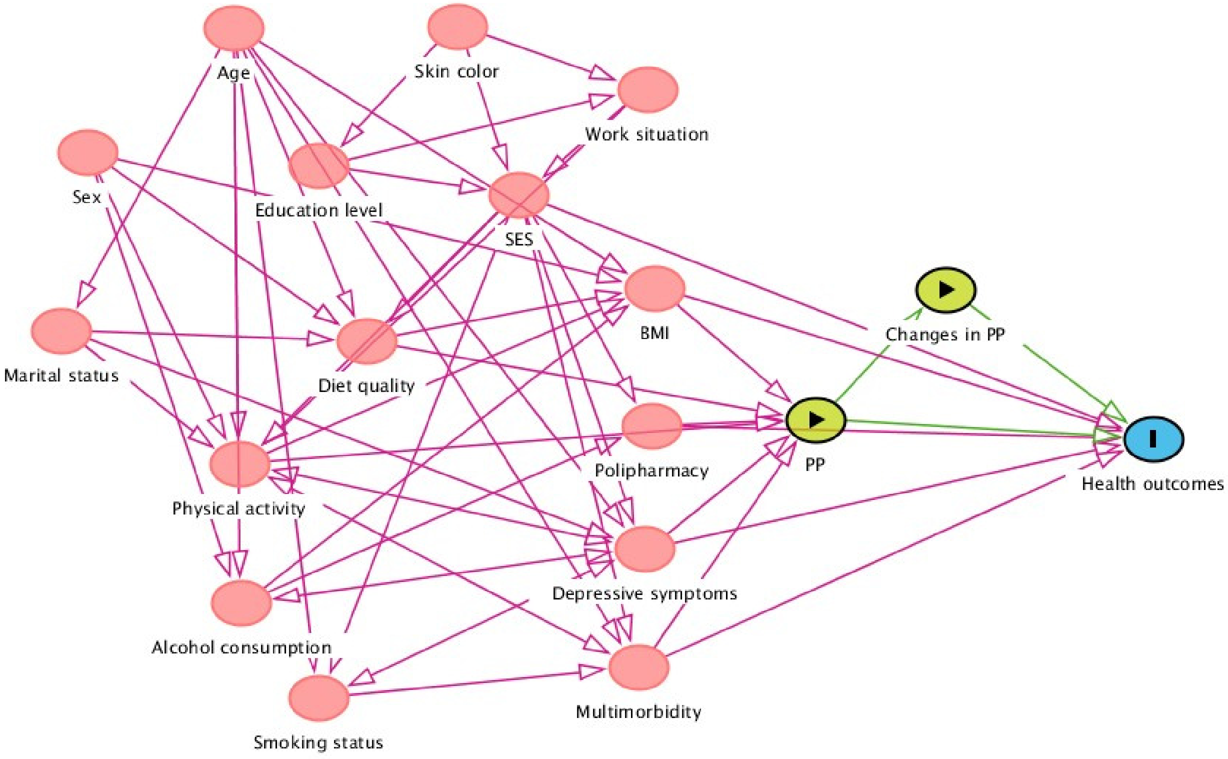

Figure S1: Adjustment model including sociodemographic, behavioral and health variables used for statistical analysis. Colors: pink=potential confounders (SES=socioeconomic status, BMI=body mass index); yellow=explanatory variables (PP=physical performance); blue=outcomes (negative health self-perception, consultations with health professionals, physical disability, falls, and hospitalization). Arrows: indicate the direction of the association between the variables (unidirectional or bidirectional).
